# Extracellular vesicles mediate antibody-resistant transmission of SARS-CoV-2

**DOI:** 10.1038/s41421-022-00510-2

**Published:** 2023-01-06

**Authors:** Bingqing Xia, Xiaoyan Pan, Rong-Hua Luo, Xurui Shen, Shuangqu Li, Yi Wang, Xiaoli Zuo, Yan Wu, Yingqi Guo, Gengfu Xiao, Qiguang Li, Xin-Yan Long, Xiao-Yan He, Hong-Yi Zheng, Ying Lu, Wei Pang, Yong-Tang Zheng, Jia Li, Lei-Ke Zhang, Zhaobing Gao

**Affiliations:** 1 Stake Key Laboratory of Drug Research, Shanghai Institute of Materia Medica, Chinese Academy of Sciences, Shanghai, China; 2 University of Chinese Academy of Sciences, Beijing, China; 3 State Key Laboratory of Virology, Wuhan Institute of Virology, Center for Biosafety Mega-Science, Chinese Academy of Sciences, Wuhan, Hubei China; 4 Key Laboratory of Animal Models and Human Disease Mechanisms of Chinese Academy of Sciences and Yunnan Province, Kunming Institute of Zoology, Chinese Academy of Sciences, Kunming, Yunnan China; 5 Public Technology Service Center, Kunming Institute of Zoology, Chinese Academy of Sciences, Kunming, Yunnan China; 6 Kunming College of Life Science, University of Chinese Academy of Sciences, Kunming, Yunnan China; 7 Zhongshan Institute for Drug Research, Institution for Drug Discovery Innovation, Chinese Academy of Science, Zhongshan, Guangdong China; 8 School of Pharmacy, Fudan University, Shanghai, China

**Keywords:** Golgi, Super-resolution microscopy

## Abstract

Severe acute respiratory syndrome coronavirus 2 (SARS-CoV-2) has caused a global pandemic. Antibody resistance dampens neutralizing antibody therapy and threatens current global Coronavirus (COVID-19) vaccine campaigns. In addition to the emergence of resistant SARS-CoV-2 variants, little is known about how SARS-CoV-2 evades antibodies. Here, we report a novel mechanism of extracellular vesicle (EV)-mediated cell-to-cell transmission of SARS-CoV-2, which facilitates SARS-CoV-2 to escape from neutralizing antibodies. These EVs, initially observed in SARS-CoV-2 envelope protein-expressing cells, are secreted by various SARS-CoV-2-infected cells, including Vero E6, Calu-3, and HPAEpiC cells, undergoing infection-induced pyroptosis. Various SARS-CoV-2-infected cells produce similar EVs characterized by extra-large sizes (1.6–9.5 μm in diameter, average diameter > 4.2 μm) much larger than previously reported virus-generated vesicles. Transmission electron microscopy analysis and plaque assay reveal that these SARS-CoV-2-induced EVs contain large amounts of live virus particles. In particular, the vesicle-cloaked SARS-CoV-2 virus is resistant to neutralizing antibodies and able to reinfect naïve cells independent of the reported receptors and cofactors. Consistently, the constructed 3D images show that intact EVs could be taken up by recipient cells directly, supporting vesicle-mediated cell-to-cell transmission of SARS-CoV-2. Our findings reveal a novel mechanism of receptor-independent SARS-CoV-2 infection via cell-to-cell transmission, provide new insights into antibody resistance of SARS-CoV-2 and suggest potential targets for future antiviral therapeutics.

## Introduction

Severe acute respiratory syndrome coronavirus 2 (SARS-CoV-2) explosively spreads and has clinical manifestations ranging from asymptomatic infection to respiratory failure and even death^1–3^. SARS-CoV-2 is a novel virus belonging to the *Beta coronavirus* genus and exhibits high similarities to another two coronaviruses, severe acute respiratory syndrome coronavirus (SARS-CoV) and Middle East respiratory syndrome coronavirus (MERS-CoV), which have caused large-scale outbreaks over the past two decades^4,5^. Previous studies found that the SARS-CoV-2 lifecycle commences with classic binding of the spike (S) protein to its cognate receptor on the surface of the host cell, human angiotensin-converting enzyme 2 (hACE-2). The cleavage of the S1/S2 site by the membrane protease serine 2 (TMPRSS2) and virus–cell membrane fusion mediated by lysosomal cathepsin L determine the efficiency of virus entry^6–9^. Single-cell sequencing data exhibit that the hACE-2 receptor is expressed in less than 1% cells in certain tissues at low expression levels, including heart, liver, brain, lung, and trachea, yet SARS-CoV-2 RNA may still be detected in these organs^10–13^. Notably, SARS-CoV-2 causes profound and severe pulmonary damage, but hACE-2 expression in lung cells is lower than that in some extrapulmonary tissues^14–16^. Additionally, the expression of hACE-2 was not upregulated in the lungs of COVID-19 patients^17^. Some researchers therefore hypothesize that there may still be unappreciated pathways for SARS-CoV-2 entry into host cells. Two recent studies found that an additional factor, neuropilin-1 (NRP-1), which serves as the furin cleavage substrate of the S protein, could enhance the infectivity of SARS-CoV-2^18,19^. In addition, a recent work showed that a novel candidate receptor, the tyrosine-protein kinase receptor UFO (AXL), specifically interacted with SARS-CoV-2 S protein and promoted viral entry as efficiently as hACE-2 overexpression via “apoptotic mimicry”. Downregulating AXL was found to reduce SARS-CoV-2 infection of pulmonary cells^11^. These vital findings not only deepen our understanding of the transmission of SARS-CoV-2 but also provide new perspectives for developing anti-SARS-CoV-2 drugs and antibodies.

Neutralizing antibodies have been proposed as a cutting-edge antiviral strategy in the therapeutic race against SARS-CoV-2^20^. Current antibody therapies are divided into anti-viral and anti-inflammatory treatments. Among antibody options, convalescent plasma (CP) treatment is receiving significant attention, which may provide patients with immediate passive immunity^21,22^. However, CP therapy is suboptimal and fails to reverse respiratory failure and reduce mortality^23,24^. Another promising treatment option was monoclonal antibodies designed to mainly target the S protein of the virus membrane or the hACE-2 receptor of the host cell plasma membrane, thereby preventing viral binding with its receptor. To date, at least eight antibody candidates targeting the S protein have entered different stages of clinical studies^20^. The LY-CoV555 antibody from Lilly was the first neutralizing antibody to receive FDA emergency use authorization for the treatment of COVID-19. In a phase II trial, LY-CoV555 appeared to accelerate the natural decline in viral load at day 2 in outpatients diagnosed with mild or moderate COVID-19, but did not have the same effect in patients with severe COVID-19 or those with prolonged illness^25,26^. However, LY-CoV555 exhibited an unsatisfactory therapeutic effect on SARS-CoV-2 variant B.1.1.7^27^. In particular, the “cocktail antibodies” BRII-196 plus BRII-198, developed against variants, were stopped early due to lack of utility^28^. Appearance of spontaneous mutations in SARS-CoV-2 is the main reason for the unsatisfactory effect of neutralizing antibodies targeting the virus. SARS-CoV-2 variants may dampen the efficacy and specificity of antibodies and further lead to new viral strains that may gradually develop resistance to existing antibodies^29,30^. Thus, the problem of SARS-CoV-2 escaping from antibodies needs much effort to be solved.

## Results

### The SARS-CoV-2 envelope protein induces extracellular vesicles containing virus particles

Our previous research has demonstrated that the SARS-CoV-2 structural envelope (2-E) protein forms a type of pH-sensitive cation channel, and that heterogeneous expression of 2-E channels causes host cell death^31^. After 2-E transfection, we found that the cell swelling and membrane protrusion began at 16 h, followed by extracellular vesicle (EV) secretion from mother cells. At approximately 24 h, the cell membrane exhibited an inflated ‘balloon-like’ appearance prior to its rupture (Supplementary Figs. S1, S2a and Video S1). The swelling ‘balloon-like’ bubbles at the end of the cell death process is a typical feature of necrosis including necroptosis and pyroptosis^32–35^. Among the tested necrotic hallmarks, gasdermin E (GSDME) was found to be cleaved into two fragments upon 2-E expression in all three cell lines (Vero E6, A549, and Hela), concurrent with activation of caspase-3 and -7 (Supplementary Figs. S2, S3). Combined with Annexin V (AV) and propidium iodide (PI) staining examination, these above results suggest that GSDME cleavage leads to pyroptosis during 2-E-mediated cell death^33^.

**Fig. 1:**
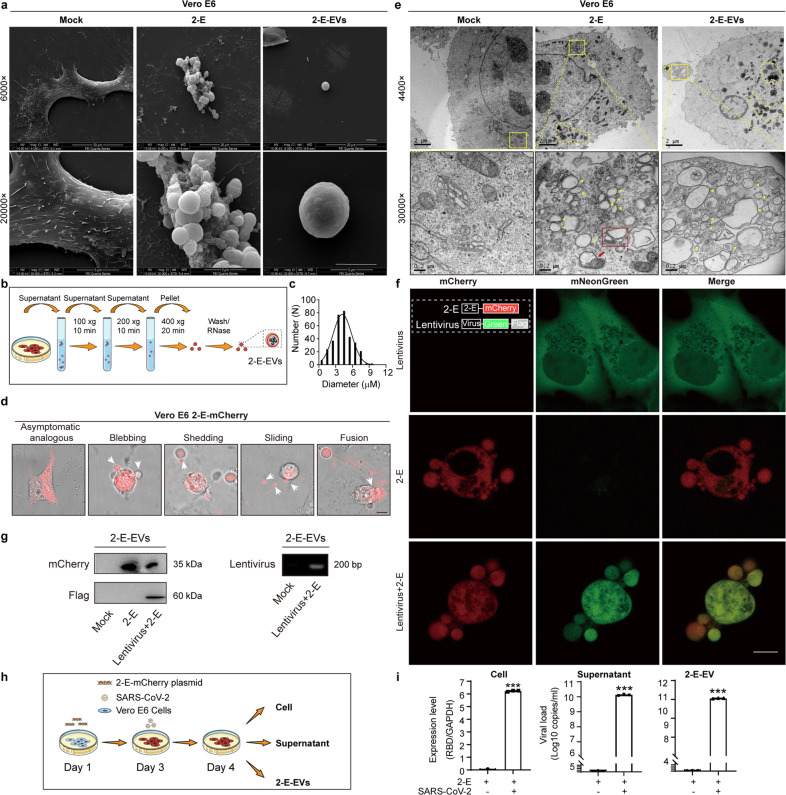
The 2-E protein induces secretion of vesicles containing virus particles. **a** SEM images of 2-E-transfected Vero E6 cells and EVs isolated from the transfected cells. Scale bar, 5 μm. **b** The flow chart showing the isolation of 2-E-EVs. **c** Diameter variation of 2-E-EVs. **d** Microscopy images of 2-E-mCherry-expressing Vero E6 cells during cell death. Five stages of 2-E-mCherry-transfected cells are shown. White arrows indicate 2-E-EVs. Scale bar, 10 μm. **e** TEM images of 2-E-transfected Vero E6 cells and 2-E-EVs. Yellow triangles, SMVs; red box, swelling Golgi; red arrows, damaged mitochondria; yellow dotted circles, lysosomes. Scale bars, 2 μm (up), 0.2 μm (bottom). **f** Microscopy images of Vero E6 cells. The cells were infected with a lentivirus vector (Lenti-mNeonGreen-3×Flag) and then transfected with 2-E-mCherry plasmid to induce vesicles. Scale bar, 10 μm. Illustration in the left corner shows a schematic view of the virus and plasmids used. An mCherry (red) and an mNeonGreen (green) tag were added to the C-termini of 2-E and lentivirus, respectively. **g** Left: immunoblot showing mCherry and Flag signals in isolated 2-E-EVs; all lanes were loaded with the same amount of total proteins. Right: nucleic acid gel image showing lentivirus in isolated 2-E-EVs. Shown is one representative experiment of three. **h** Schematic representation of infection experiments. Vero E6 cells were first transfected with the 2-E-mCherry plasmid and then infected with SARS-CoV-2. The next day, the cells, supernatant and 2-E-EVs were collected. **i** qRT-PCR analysis of SARS-CoV-2 copies after transfection with the 2-E-mCherry plasmid and infection. The histograms show viral copies in cells (left), supernatant (middle), and isolated 2-E-EVs (right). Data are means ± SEM of at least three independent experiments. *P < 0.05, **P < 0.01, ***P < 0.001; unpaired Student’s *t*-test.

Interestingly, a number of EVs with unknown biological functions were observed to be secreted by Vero E6 cells expressing 2-E under scanning electron microscopy (SEM) (Fig. 1a). SEM showed that the 2-E-expressing cell bodies became round, accompanied by partial detachment of the cell from the culture slide. Many EVs protruded from the host cells and attached to the surface, with similar appearance to bunches of grapes. The shed EVs were round, smooth, and smaller than their mother cells (Fig. 1a). Each blebbing cell could produce 1–15 EVs (3 on average). With reference to the separation parameters of cell organelles such as mitochondria and the endoplasmic reticulum (ER), 2-E-induced EVs (2E-EVs) were isolated using differential centrifugation after multiple rounds of optimization^36,37^. The size of 2-E-EVs was confirmed by measuring the cross-sectional diameters of 100 randomly selected EVs under microscope; 72% of the vesicles were between 3 and 6 μm in diameter, with an average diameter of 4.3 μm (Fig. 1b, c; Supplementary Fig. S4).

To understand how 2-E stimulates the secretion of EVs, the blebbing process was first visualized in Vero E6 cells transfected with 2-E-mCherry plasmids (Fig. 1d; Supplementary Fig. S5 and Videos S1–S3). The videos revealed three major characteristics of these EVs, including shedding, sliding and fusion, implying that the EVs may traffic from their mother cells to other cells within their immediate microenvironment as well as at a distance. Transmission electron microscopy (TEM) was used to further investigate the inside of EVs. After 2-E protein expression, the ultrastructure of cells underwent tremendous changes. First, a large number of single-membrane vacuoles (SMVs) appeared. These SMVs could be divided into two subgroups with diameters of 79.9 ± 31.2 and 252.2 ± 148.9 nm, respectively (Fig. 1e, yellow triangles). Second, the number of lysosomes increased significantly in cells (Fig. 1e, yellow dotted circle). Last, ER, mitochondria and Golgi swelled. Closer inspection showed that the Golgi and mitochondria linked several vacuoles together (Fig. 1e, red box and red arrow). Amazingly, the shedding EVs have ultrastructural characteristics similar to those of their mother cells. As shown in the right panel of Fig. 1e, a number of SMVs, including both large and small SMVs, appeared in a representative EV simultaneously (Fig. 1e, yellow triangles). Additionally, many lysosomes were also observed inside the EVs (Fig. 1e, yellow dotted circles).

It has been previously shown that SARS-CoV-2 may use lysosomes for egress^38^. We speculated that 2-E-EVs could package viruses as well. To explore this hypothesis, a lentivirus with a green fluorescence tag (Lenti-mNeonGreen-3×Flag) was first constructed as a viral simulation vector. We infected Vero E6 cells with the lentivirus and then transfected 2-E-mCherry plasmids to induce EVs. Vero E6 cells transfected with 2-E plasmids alone were used as controls. As shown in Fig. 1f, we found that the red EVs indeed wrapped green viral particles, suggesting that mature lentivirus particles were secreted from the mother cell into the EVs (Fig. 1f). Consistently, a high level of lentivirus (anti-Flag) was detected in the red EVs isolated from the lentivirus infection group. In contrast, EVs isolated from the control group contained 2-E protein (anti-mCherry) only (Fig. 1g, left). PCR using lentivirus-specific primers also supported the conclusion that EVs packaged lentiviruses (Fig. 1g, right). Whether 2-E-EVs could package SARS-CoV-2 was then investigated. Vero E6 cells were transfected with 2-E-mCherry plasmids and then infected with SARS-CoV-2 (Fig. 1h). The SARS-CoV-2 levels in the cell, supernatant and isolated EVs were examined using quantitative real-time PCR (qRT-PCR) recognizing the SARS-CoV-2 receptor-binding domain (RBD). The viral copies in the EVs were found to be more than 1 × 10^11^ viral copies/mL (Fig. 1i), confirming that the 2-E-EVs could package SARS-CoV-2.

### SARS-CoV-2 infection produces extracellular vesicles

**Fig. 2:**
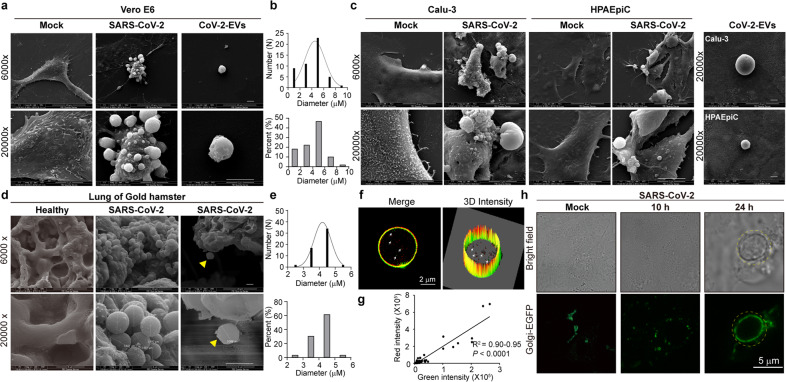
SARS-CoV-2 induces secretion of extracellular vesicles. **a** SEM images of Vero E6 cells infected with SARS-CoV-2 (MOI = 1). **b** Diameters of vesicles derived from SARS-CoV-2-infected Vero E6 cells. Data are the means ± SEM. **c** SEM images of cells and EVs derived from Calu-3 (MOI = 2) and HPAEpiC cells (MOI = 5) infected with SARS-CoV-2. **d** SEM images of healthy golden hamster lung tissues and those infected with SARS-CoV-2. Yellow triangles, a shed vesicle from the damaged lung. **e** Diameter range of vesicles derived from SARS-CoV-2-infected golden hamster lung tissues. **f** Images of isolated 2-E-EVs showing co-transfected 2-E-mCherry and Golgi-EGFP (B4GALT1-EGFP). 2-E-mCherry is co-localized with the Golgi marker on 2-E-EV membranes. Interspersed Golgi apparatus were wrapped inside the isolated EVs (white arrows). **g** Linear regression analysis showing the correlation between red and green signals. **h** Vero E6 cells were transfected with Golgi-EGFP and then infected with SARS-CoV-2 (MOI = 1) (right). Images were captured at the indicated times after infection. Scale bars, 2 μm (**f**), 5 μm (**a**, **c**, **d**, **h**).

Then, whether SARS-CoV-2 infection could produce EVs was inspected in various cells. We found that the plasma membrane of SARS-CoV-2-infected Vero E6 cells also underwent swelling. Subsequently, the cell body rounded up and progressed toward blebbing (Fig. 2a). More than five EVs protruded from each infected mother cells and attached to the surface, which were similar in shape to bunches of grapes. Using a method similar to that used to isolate 2-E-EVs, SARS-CoV-2-induced EVs (CoV-2-EVs) were isolated and then measured, and 81% of the vesicles had a diameter between 3 and 5 μm, with an average diameter of 4.9 μm (Fig. 2b). Interestingly to note, the size of CoV-2-EVs is much larger than those of other previously reported viral-generated vesicles, such as exosomes (0.04–0.15 μm), microvesicles (0.05–1 μm) and apoptotic bodies (0.5–2 μm)^39–41^. Notably, similar protruding or shedding EVs were also observed in the infected human airway epithelial cell line Calu-3 and human pulmonary alveolar epithelial cell line HPAEpiC (Fig. 2c). In addition, we examined SARS-CoV-2-infected lungs of golden hamsters, a widely used experimental animal model of SARS-CoV-2 infection. Golden hamsters were infected intranasally with 10^4^ 50% tissue culture infective dose (TCID50) of SARS-CoV-2 or DMEM medium as control. At Day 3 post infection (3 dpi), the lungs were collected to monitor viral replication and histopathological changes. Viral load in the lungs reached 10^6^ copies/μg RNA at 3 dpi. We observed mononuclear cell infiltration in the regions where viral antigen was detected at 3 dpi. Clusters of viruses were detected in the swollen and shedding tissues (Supplementary Fig. S6). Then, different sections of each sample were examined using SEM. Interestingly, numerous vesicle structures were docked at the extracellular side of bronchovascular and alveolar network, while no obvious changes were observed in the control group (Fig. 2d, *n* > 3). Crucially, the shed vesicles from the damaged lung were also captured (right column, yellow triangle). 90% of the vesicles show cross-sectional diameters between 3.5 and 5.5 μm, with an average diameter of 4.2 μm, which was close to the diameter of the EVs observed in vitro (Fig. 2e). Notably, a recent research also reported that EVs with different sizes were detected in the blood of COVID-19 patients and are related to the severity of the disease^42^. Together, these results support that EVs are physiopathologically relevant in SARS-CoV-2 infection.

In addition to the plasma membrane, the endomembrane system, which includes the nuclear membrane, lysosomes, Golgi complex, and ER, is also essential to the function of the cell. Among them, both the ER and Golgi have been reported to participate in the life cycle of SARS-CoV-2^43^. To explore the potential origin of these EVs, three subcellular membrane markers, plasma-EGFP, ER-EGFP, and Golgi-EGFP, were first co-transfected with 2-E plasmids. Of the three markers, the membranes of 2-E-EVs were strongly positive for the Golgi marker and spot positive for the plasma membrane marker. Quantitative analysis confirmed that the isolated EVs were composed of Golgi membrane and were co-localized with 2-E (> 80%) (Fig. 2f, g; Supplementary Fig. S7). We further investigated the fate of the Golgi membrane during EV production and secretion. At the early stage (10–12 h after transfection), 2-E-mCherry mainly accumulated in an unidentified perinuclear compartment. At approximately 16 h, Golgi marker-labeled EVs emerged from the cell surface and were rapidly released (Supplementary Fig. S8). Thus, Golgi membranes were secreted and contributed to the formation of 2-E-EVs. Similar to the observation in the 2-E-EVs, the membranes of CoV-2-EVs were also positive for the Golgi-EGFP, suggesting that the Golgi membranes contributed to the formation of these EVs (Fig. 2h). Further lipid analysis would be helpful to determine the origin of these EVs.

### SARS-CoV-2-induced vesicles contain a large number of virus particles

**Fig. 3:**
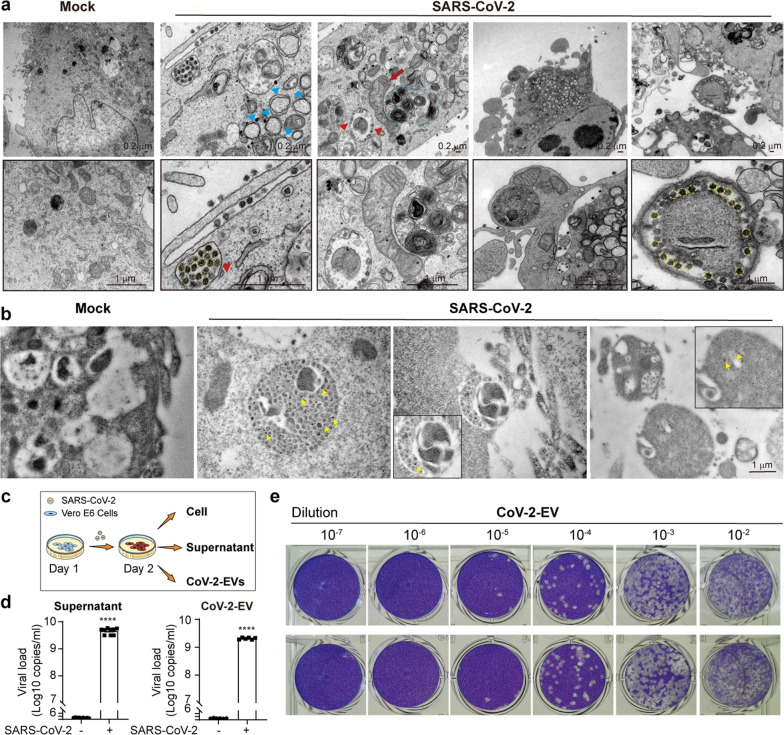
CoV-2-EVs contain a large amount of virus particles. **a** TEM images of Vero E6 cells infected with SARS-CoV-2. Blue triangles, double-membrane vesicles (DMVs); red triangles, SMVs; yellow dotted circles, virus particles; red arrow, damaged mitochondria; blue dotted circle, lysosomes (MOI = 1). **b** Immunoelectron microscopy analyses of infected Vero E6 cells using SARS-CoV-2 nucleocapsid immunogold labeling (MOI = 1). Yellow arrows, virus particles. **c** Schematic representation of infection experiments. Vero E6 cells were infected with SARS-CoV-2. On the next day, the cells, supernatant, and CoV-2-EVs were collected. **d** qRT-PCR analysis of SARS-CoV-2 RNA in supernatant and isolated EVs from SARS-CoV-2-infected Vero E6 cells. **e** Plaque reduction assay of CoV-2-EVs. Data are the means ± SEM. *P < 0.05, **P < 0.01, ***P < 0.001, ****P < 0.0001; unpaired Student’s *t*-test.

Ultrastructural analysis of SARS-CoV-2-infected cells and secreted EVs was further performed using TEM. The Golgi, mitochondria and ER were also altered in SARS-CoV-2-infected cells, with swelling and increased intracristal space and matrix density (Fig. 3a, red arrows)^44,45^. Multiple characteristic visual fields for CoV-2-EVs were captured. First, there were numerous dense virus particles and mitochondria in a shedding CoV-2-EV. Second, a large number of SARS-CoV-2 virions were encapsulated in the shed CoV-2-EVs (Fig. 3a, yellow dotted circles). These virions displayed an average diameter of 75 ± 10 nm, consistent with previous reports^43^. To further verify the presence of SARS-CoV-2 virions in CoV-2-EVs, we performed immunohistochemistry analyses using SARS-CoV-2 nucleocapsid immunogold labeling. SARS-CoV-2-infected (MOI = 1) Vero E6 cells showed strong labeling for the nucleocapsid in the cytosol and in viral particles that accumulated intracellularly (Fig. 3b). In shed CoV-2-EVs, in addition to cellular contents, viral particles were visible and marked by gold particles (Fig. 3b, yellow arrows). Plaque-reduction assay and qRT-PCR also supported that these CoV-2-EVs contained a large number of infectious viruses, as high as 2.3 × 10^7^ PFU/mL and 2 × 10^9^ viral copies/mL (Fig. 3c–e). We noticed that the virial copies in the 2-E-EVs were higher than those in the virus-induced EVs, implying that redundant 2-E proteins may facilitate virus production, packaging, or EV secretion.

### SARS-CoV-2-induced vesicles help viruses escape from neutralizing antibodies and establish a productive infection

**Fig. 4:**
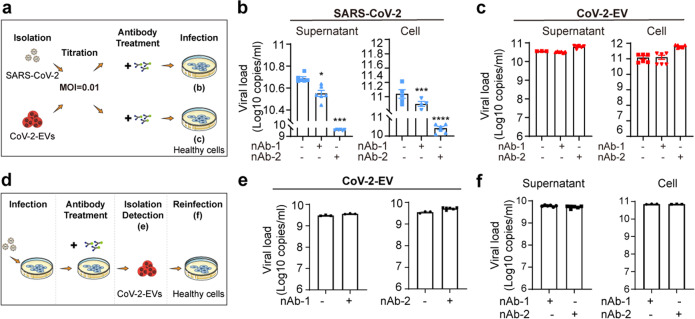
CoV-2-EVs facilitate SARS-CoV-2 escape from neutralizing antibodies and establish a productive infection. **a** Schematic representation of SARS-CoV-2 and CoV-2-EV infection experiments. **b**, **c** qRT-PCR analysis of SARS-CoV-2 levels in cells and supernatant after infection by viral particles (**b**) and CoV-2-EVs (**c**) pretreated with nAb-1 or nAb-2. **d** Schematic representation of secondary infection experiments. Vero E6 cells were infected with live SARS-CoV-2 virus for 1 h, and then neutralizing antibodies or control IgG proteins were added. CoV-2-EVs were isolated from the supernatant by differential centrifugation as described in Fig. 1b. Naïve Vero E6 cells were incubated with CoV-2-EVs for 24 h and then replaced with fresh medium. Then, the viral copies were tested by qRT-PCR. **e** qRT-PCR analysis of viral copies in CoV-2-EVs from **d**. **f** qRT-PCR analysis of SARS-CoV-2 levels in cells and supernatant after secondary infection by CoV-2-EVs pretreated with nAb-1 or nAb-2. Data are the means ± SEM of three independent experiments. *P < 0.05, **P < 0.01, ***P < 0.001, ****P < 0.0001. Analysis was performed using one-way ANOVA.

Some earlier studies reported that EVs, such as exosomes can protect viruses from the neutralizing antibodies^46,47^. Whether CoV-2-EVs could carry out similar functions was then examined. Two reported neutralizing antibodies against SARS-CoV-2 were used as a proof of concept. The first is a recombinant monoclonal neutralizing antibody from HEK293 cells, which recognizes recombinant spike RBD-mFc protein (nAb-1)^48^. The other is RBD-specific immunoglobulin F(ab’) 2 fragment, obtained from hyperimmune equine plasma (nAb-2)^49^. The neutralizing efficiencies of these two antibodies were verified in vitro. SARS-CoV-2 was incubated with Vero E6 cells for 1 h and then removed by replacement with fresh medium. Under the 0.01 MOI live virus infection conditions, both nAb-1 and nAb-2 antibodies were able to suppress SARS-CoV-2 production, based on the viral inhibition in supernatant with EC_50_ values of 0.59 and 0.05 μg/mL, respectively (Supplementary Fig. S9). To evaluate potential EV-mediated antibody resistance, two sets of experiments were designed and carried out. In the first set of experiments, to strictly control the infection conditions, the neutralization efficiency of the two antibodies for free and vesicle-encapsulated viruses was evaluated under equal-titer infection (MOI = 0.01) (Fig. 4a). Interestingly, although the infection was largely suppressed by nAb-1 or nAb-2 in the free virus groups (Fig. 4b), neither nAb-1 nor nAb-2 suppressed the infection of healthy cells in the CoV-2-EV groups (Fig. 4c). In the second set of experiments, to simulate the antibody therapy process, neutralizing antibodies were added to culture plates 2 h after infection. The CoV-2-EVs were isolated 24 h after infection, and the viral copies in the CoV-2-EVs were examined (Fig. 4d). Both antibodies showed negligible inhibitory effects on live virus levels in CoV-2-EVs, and the antibody-treated CoV-2-EVs retained reinfection ability (Fig. 4e, f). In addition to mutations, it has been reported that S protein-dependent cell-to-cell transmission may also dampen the efficacy of SARS-CoV-2 antibody^50^. Although more in vivo evidence is needed, our results demonstrate that CoV-2-EVs can protect the virus against neutralizing antibodies, suggesting a novel mechanism by which SARS-CoV-2 escapes from antibody neutralization.

### CoV-2-EVs mediate the entry of SARS-CoV-2 into cells independent of known receptors of recipient cells

**Fig. 5:**
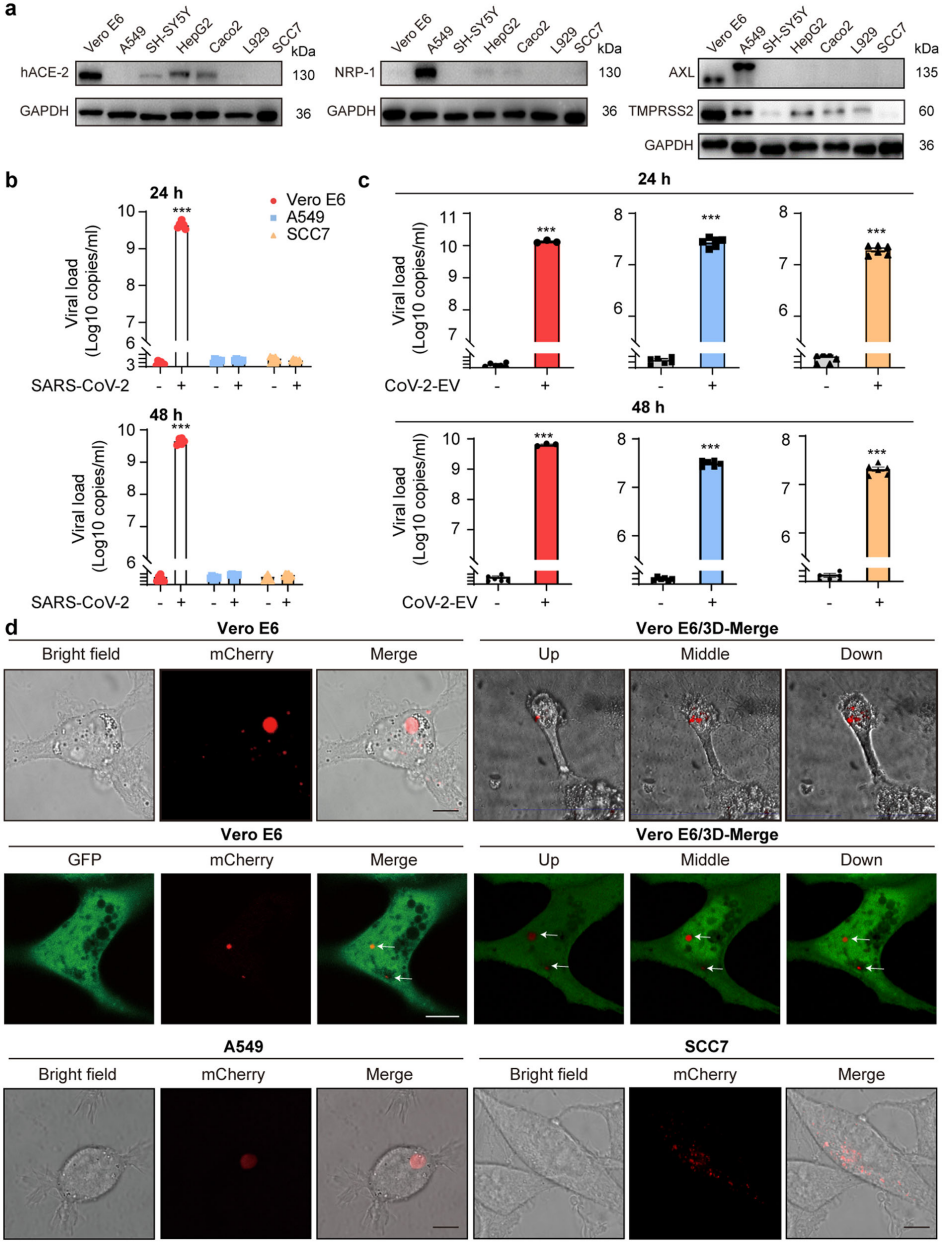
CoV-2-EVs mediate the entry of SARS-CoV-2 into cells independent of known receptors and cofactors on host cells. **a** Expression levels of hACE-2, NRP-1, AXL and TMPRSS2 in different cell lines. **b** Supernatant viral copies of Vero E6, A549 and SCC7 cells after infection with SARS-CoV-2 for 24 and 48 h, respectively. Results are from more than 3 independent experiments. **c** Vero E6, A549 and SCC7 cells were incubated with isolated CoV-2-EVs, and supernatant viral copies were quantified by qRT-PCR. Results are from more than 6 independent experiments. **d** Images of Vero E6, A549 and SCC7 cells that were exposed to 2-E-mCherry-induced vesicles. Scale bars, 10 μm. Data are the means ± SEM of three independent experiments. *P < 0.05, **P < 0.01, ***P < 0.001. Analyses were performed using one-way ANOVA (**b**) or unpaired Student’s *t*-test (**c**).

Although it is logical that EVs could shelter SARS-CoV-2 from their neutralizing antibodies through impermeable membranes, one may argue that EVs may function as temporal reservoirs only and the virus continues to be released from those disrupted EVs to infect naïve cells via classic virus–receptor pathways^7,8,18,19^. Whether EVs represent a potential pathway for SARS-CoV-2 entry and infectivity was investigated. The expression levels of hACE-2, AXL, NRP-1, and TMPRSS2, the reported major receptors and cofactors for SARS-CoV-2, were first screened in seven cell lines. Three cell lines were chosen for further exploration: Vero E6, a *Cercopithecus aethiops* kidney epithelial cell line, expressing hACE-2, TMPRSS2, and AXL but not NRP-1; A549, a human alveolar epithelial cell line, expressing AXL, NRP-1, and TMPRSS2 but not hACE-2; and SCC7, a mouse skin cancer cell line, with no detectable hACE-2, AXL, NRP-1 or TMPRSS2 expression (Fig. 5a). These cell lines were incubated with free virus particles or isolated CoV-2-EVs. We found that hACE-2 rendered Vero E6 cells susceptible to free virus infection, whereas the other two cell lines lacking hACE-2 expression were insensitive to free virus particles (Fig. 5b). In contrast, all three cell lines were infected with isolated CoV-2-EVs, regardless of whether they express hACE-2, AXL, NRP-1 or TMPRSS2 (Fig. 5c). The viral titers of the infected Vero E6 cells by CoV-2-EVs were ~2.3 × 10^5^ PFU/mL and 1.7 × 10^6^ PFU/mL for 24 and 48 h, respectively, after infection (Supplementary Fig. S10).

To validate that CoV-2-EVs mediate viral infection independent of receptors, the pulmonary cell line H1299 was selected for further experiments. The H1299 cell line is rich in AXL expression but lacks hACE-2 expression. Wild-type and AXL^–/–^ H1299 cells were treated with isolated CoV-2-EVs, and the viral copies in the supernatant were measured 24 h after the treatment. We found that both wild-type and AXL^–/–^ cells were effectively infected (Supplementary Fig. S11a, b). These data indicate that CoV-2 EVs containing living viruses can reinfect naïve cells independent of recipient cell receptors.

How EVs enter recipient cells was then explored. Due to the deficiency of imaging equipment and fluorescence-tagged virus in our P3 laboratory, we used 2-E-mCherry-induced EVs to explore the potential mechanism underlying EV-mediated transmission in a general biological laboratory. Vero E6, A549, and SCC7 cell lines were first exposed to red EVs isolated from 2-E-mCherry-transfected Vero E6 cells for 24 h. Increased cytosolic fluorescence, which relates to the quantity of EVs taken up by the recipient cells, was observed during incubation (Fig. 5d, up row). To improve the visual effects, in a parallel experiment, naïve Vero E6 cells were first transfected with EGFP plasmids and then exposed to red EVs (Fig. 5d, middle row). The constructed 3D images and videos supported the presence of intact EVs in cells (Fig. 5d; Supplementary Videos S4, S5). The incorporation of 2-E proteins in the recipient cells was examined by western blotting. Consistent with the images, 2-E-mCherry proteins were enriched in the EV-treated cells compared with the untreated cells (Supplementary Fig. S12). Similar phenotypes were also observed in the pulmonary cell line H1299 when it was treated with red EVs (Supplementary Fig. S11c, d). These results indicate that intact EVs can be taken up directly by recipient cells, suggesting a vesicle-mediated cell-to-cell transmission of SARS-CoV-2.

## Discussion

Vaccines and neutralizing antibodies are most promising preventive tactics and effective treatments to end the global healthy crisis caused by SARS-CoV-2. However, viruses behave as soldiers fighting against our suppression strategy through various means^20,29,30^. Here, we report a vesicle-mediated cell-to-cell transmission of SARS-CoV-2, which facilitates virus escape from neutralizing antibodies.

In our previous study, we demonstrated that 2-E mediates cell death by forming cation channels, whereas the mechanism by which this occurs is unknown^31^. In this study, we showed that the pyroptosis-related protein GSDME is cleaved after 2-E expression, which targets the plasma membrane to induce cell death. GSDME belongs to the gasdermin superfamily, which has recently been implicated as a key mediator of chemical-induced pyroptosis and cell disassembly downstream of apoptotic caspase-3. The GSDME-dependent pyroptosis was also observed in vesicular stomatitis virus infection^51^. Secondary necrosis, including necroptosis and pyroptosis has long been regarded as a terminal event following the completion of the apoptotic programmed cell death and is considered to be nonspecific and nonprogrammed osmotic swelling^33,52^. However, in addition to the stimulations mentioned above, whether and what other factors could lead to secondary pyroptosis remain unknown. Our results show that the 2-E-induced secondary pyroptosis is orchestrated by the activity of apoptotic caspase-3, which cleaves GSDME to produce necrotic fragments that might target and penetrate the plasma membrane. Our results support the conclusion that viral channel 2-E is an effective stimulator for the GSDME-dependent pyroptosis.

During the 2-E-induced pyroptosis, the production of EVs was observed. The EV membrane contains a large amount of Golgi membrane and a bit of plasma membrane, which might be due to the membrane rearrangement and fusion during the process of cell death^53,54^. We experimentally demonstrated that EVs induced by SARS-CoV-2 could package large numbers of viruses. It has been widely accepted that non-enveloped viruses, such as *poliovirus*, *coxsackievirus*, and *rhinovirus*, are always transmitted via vesicles^40,55^. Generally, being an enveloped RNA virus, SARS-CoV-2 is largely thought to behave as discrete infectious units during transmission^7,56^. Upending this view, our study is the first to report that SARS-CoV-2 can travel between cells, not only as independent viral particles but also as clusters of viral particles within EVs. Remarkably, the diameters of CoV-2-EVs range from 1 to 10 μm, displaying an average diameter of 4.9 μm, which is much larger than those of other virus-generated vesicles^40,57^. In contrast, the SARS-CoV-2 particle is only ~80 nm^58^. EV with a diameter of 1 μm has been found to greatly enhance the transmission and infection efficiency of *Marseillevirus*^59^. We propose that CoV-2-EVs might act as a trunk for the virus and perhaps promote highly efficient propagation of SARS-CoV-2.

In addition to physically protecting viruses from neutralizing antibodies, vesicle-mediated transmission may indicate a new perspective for the emergence of SARS-CoV-2 antibody resistance. Virus quasispecies is a process of virus self-correction and natural selection to retain high-quality sequences, ensuring offspring survival, whereas mutation is a natural byproduct of viral replication and genetic cooperativity^60^. Due to the rapid replication kinetics, viral RNA-dependent RNA polymerases (RdRps) act too late to correct errors in their genome, and each RNA genome differs from one another in terms of replicative fitness in the next infection cycle^41,60,61^. According to these theories on quasispecies and genetic cooperation among viruses, CoV-2-EVs carrying an excessive amount of virus particles may help rapid viral antigenic evolution^40,62^. Antigenic evolution is thought to be the main reason for SARS-CoV-2 eluding antibodies and the reduction in vaccine efficiency^29,30^. CoV-2-EVs contain a large mix of genomes, perhaps providing more opportunities for SARS-CoV-2 to freely match and produce different variants^60,63^. Indeed, it has been found that the evolution rate of SARS-CoV-2 was abnormal and that its genome has changed greatly from its original sequence^64^. Some newly identified mutations, such as N501Y, facilitate viruses to bind hACE-2 and spread more readily between people, especially children^65^. In addition, some variants, such as K417N, E484K, and N501Y, have been found to reduce or abolish the efficiency of 14 vaccine-elicited antibodies^66^.

In addition, it is generally accepted that the entry of SARS-CoV-2 into cells depends on a virus–receptor interaction. However, the adaptable virus can also attack tissues, such as respiratory, olfactory epithelial cells and monocytes and macrophages, that have low expression of hACE-2 and other factors common for viral entry^7,16,18,19,67^. Virus-generated EVs were discovered decades ago and play important roles in cell-to-cell communication^68,69^. In addition to viruses, these EVs can also carry a range of nucleic acids and proteins that may have a significant impact on the phenotype of recipient cells^68,69^. How the proteins and nucleic acids carried by CoV-2-EVs affect virus transmission and disease progression is worth further investigation. For the phenotypic effect to occur, the vesicles need to fuse with target cell membranes, either directly with the plasma membrane or with the endosomal membrane after endocytic uptake^69^. The molecular basis underlying the entry of CoV-2-EVs remains unclear, it is known that vesicles produced by many cells can facilitate viral spread to other susceptible cells by endocytic processes^69,70^. Consistently, the presence of intact EVs inside cells suggests that the vesicles were taken up by the recipient cells directly. Our study proposes a vesicle-mediated cell-to-cell transmission during SARS-CoV-2 infection and offers a novel landscape for neutralizing antibody discovery and clinical therapy development for COVID-19.

## Materials and methods

### Plasmids and lentivirus

Wild-type SARS-CoV-2-E sequences were synthesized by the Beijing Genomics Institute (BGI, China) and cloned in pcDNA5/FRT vector (Thermo Fisher, V601020, USA). 2-E with a C-terminal mCherry tag was subcloned into pcDNA3.1 (Thermo Fisher, USA) for cell transfection and imaging, using pcDNA3.1 vector as control. The lentivirus Lenti-mNeonGreen-3×Flag (pLentipLenti-CBh3FLAG-luc2-tCMVmNeonGreenF2A-Puro) was purchased from Heyuan company with the titer 6.72E+08 TU/mL (Heyuan, China). The ER-EGFP, Plasma-EGFP and Golgi-EGFP (B4GALT1-related protein) plasmids were purchased from Clontech (USA).

### Cell culture and transfection

Vero E6, A549, SH-SY5Y, HepG2, Caco2, L929, and SCC7 cells were purchased from National Collection of Authenticated Cell Cultures, China. H1299 and H1299 AXL^–/–^ cells were obtained as a gift from Prof. Xu Li (Westlake University, Hangzhou, China). Vero E6, SH-SY5Y, HepG2, Caco2, and L929 cells were grown in 90% DMEM basal medium (Gibco, USA) supplemented with 10% fetal bovine serum (Gibco, USA) and 100 units/mL penicillin/streptomycin (Gibco, USA). A549, SCC7, H1299 and H1299 AXL^–/–^ cells were grown in RPMI-1640 basal medium (Hyclone, USA) supplemented as above. All cells were cultured in 5% CO_2_ incubator at 37 °C. Lipo3000 transfection reagent (Thermo Fisher, USA) was used for transient transfection of DNA constructs according to the manufacturer’s instructions.

### Cell viability assay

Cell viability was measured using the CCK-8 kit (40203ES60, Yeasen, China). Assays were performed according to the manufacturer’s instructions. Absorbance at 450 nm was measured with Thermo Scientific Microplate Reader (Thermo Fisher, USA).

### Isolation of 2-E-EVs and CoV-2-EVs

To generate 2-E-EVs, Vero E6 cells were transfected with 2-E plasmids for 24 h. To generate CoV-2-EVs, Vero E6 cells were infected with SARS-CoV-2 (MOI = 1) for 48 h. SARS-CoV-2 (nCoV-2019BetaCoV/Wuhan/WIV04/2019) was preserved at Wuhan institute of virology, Chinese Academy of Sciences. To isolate EVs, the following optimized differential centrifugation protocol was carried out. First, the cell supernatant was centrifuged at 100× *g* for 10 min. Next, discarded the bottom sediment, collected and centrifuged the supernatant at 200× *g* for 10 min. Then collected and centrifuged the supernatant at 400× *g* for 20 min. Last, discarded the supernatant and washed the bottom sediment once with 2% FBS DMEM plus RNase. The precipitation was isolated EVs. The RNase efficiency was detected through 1% agarose gel electrophoresis.

### SEM

To prepare 2-E-EVs, the Vero E6 cells were plated in round glass coverslips and then transfected with 2-E plasmids for 24 h. To prepare CoV-2-EVs, the Vero E6 (MOI = 1), Calu-3 (MOI = 2) and HPAEpiC (MOI = 5) cells were infected with SARS-CoV-2 virus for 24 h. SARS-CoV-2-infected golden hamsters were used as the animal model for SEM analysis. Golden hamsters were purchased from Vital River Laboratory Animal Technology Co., Ltd. All animals were allowed free access to water and diet and provided with a 12 h light/dark cycle. All of the animal experiments were performed following recommendations in the Guide for the Care and Use of Laboratory Animals of Kunming Institute of Zoology (KIZ), Chinese Academy of Science. The Institutional Committee for Animal Care and Biosafety at KIZ, Chinese Academy of Science, approved works in ABSL-3 (Approval ID: SMKX--tz-20200415-03). Golden hamsters were infected intranasally with 10^4^ TCID50 of SARS-CoV-2 or DMEM medium as control. At 3 dpi, the lungs were collected for SEM analysis. SARS-CoV-2 strain was obtained from Guangdong Provincial Center for Disease Control and Prevention, Guangdong, China (NMDC number: NMDCN0000HUI), and propagated in Vero E6 cells. All infection work involving live SARS-CoV-2 was performed in the Chinese Center for Disease Control and Prevention-approved biosafety level 3 (BSL-3) laboratory of the KIZ, Chinese Academy of Science. For the cell and EV samples for SEM, both cell and EVs were spread on the glass coverslips after separated as well. The coverslips were pre-coated with poly-d-lysine (Sigma, P6407, USA) overnight in the cell incubator before use. The sample preparation methods for SEM, including cells and lungs, were as follows. First, after washed with PBS for two times, these samples were fixed in 2.5% glutaraldehyde for 16 h at 4 °C, and virus-infected cells and lungs were fixed for > 7 days. For the golden hamster samples, lungs were sliced into 100–300 μm and fixed on the coverslips. Second, after removing the glutaraldehyde, the coverslips were washed thrice with 0.1% PBS for 10 min each and then were fixed with 1% osmium acid for 1 h at room temperature. Third, the coverslips were treated with increasing concentrations of ethanol serially of 30%, 50%, 70%, 80%, 90%, 95%, and 100% for 10 min (treated with sodium sulfate anhydrous). And the coverslip was washed with 100% ethanol for twice. After critical point drying, the coverslips were pretreated with gold for 15 min before visualizing in SEM. Sections were observed and analyzed with Quanta 250 (FEI).

### TEM

The Vero E6 cells transfected with 2-E plasmids for 24 h or infected with SARS-CoV-2 virus (SARS-COV-2 strain was obtained from Guangdong Provincial Center for Disease Control and Prevention, China, and propagated in Vero E6 cells) for 24 h (MOI = 1). For transfected cells, the cells and isolated EVs were collected and fixed overnight with 2.5% glutaraldehyde at 4 °C. On the second day, the fixatives were removed, and the cells were washed by the PBS, and then fixed by 1% osmic acid at room temperature. After 1.5 h, the cells were dehydrated with 30%, 50%, 70%, 80%, 95%, and 100% ethanol for 15 min. Next, the supernatants were replaced with 100% acetone twice. Cells were infiltrated with Epon812/acetone (1:1) mixture for 2–3 h, then replaced with pure Epon812 overnight. At last, the samples were polymerized at 60 °C for 48 h. Ultrathin sections (70 nm) were obtained by using diamond knife and collected on slot grids. The grids were post-stained with 2% Uranyl acetate and lead citrate. Sections were analyzed with a Tecnai G2 Spirit Twin (FEI) plus transmission electron microscope equipped with camera (Gatan CCD). For SARS-CoV-2-infected cells, the samples were fixed for > 7 days at 4 °C using 2.5% glutaraldehyde in 0.1 M phosphate buffer (PB) (pH 7.2), and then washed with 0.1 M PB (pH 7.2) three times for 7 min. Afterward, samples were postfixed with 1% OsO_4_ for 2 h at 4 °C, and then washed with ddH_2_O three times for 7 min, followed by serial ethanol dehydration and acetone transition for 5 min, embedding in Epon 812 resin, polymerization at 60 °C for 48 h. Serial sections of 60 nm thickness were made using a Leica EM UC7 ultramicrotome. Ultrathin sections were then loaded onto 100-mesh Cu grids and double stained with 2% uranyl acetate and lead citrate before observations employing a JEM-1400 Plus transmission electron microscope at 80 kV.

### Confocal microscopy

For confocal microscopy imaging, different ways were used. For 2-E-transfected cells, Vero E6 cells were seeded as 3 × 10^4^ cells per well in glass-bottom dishes and transfected with 2-E-mCherry plasmids on the next day. After 10 h transfection, we used the 100× oil objective confocal microscopy to observe the cells, take photos, and record videos. For lentivirus infection, the Vero E6 cells were first infected with 3.36E+06 TU/mL lentivirus plus 5 μg/mL polybrene which had a green fluorescence tag (Lenti-mNeonGreen-3×Flag). On the second day, the culture medium was replaced with fresh medium. At last, the cells were transfected with 2-E-mCherry plasmids 48 h later. The 100× oil objective was used to capture images 24 h after transfection. For 2-E-EVs, the vesicles were coated on the glass slides immediately after they were separated, and were captured using 10× and 100× objectives. For 2-E-EV incubation assay, the Vero E6, A549, SCC7, H1299, and H1299 AXL^–/–^ cells were seeded in the glass-bottom dishes on the first day. Then, the 2-E-EVs were isolated from Vero E6 cells, and added into the culture media of these cells. After 24 h, cells were observed using the 100× oil objective. To explore the potential origin of 2-E-EVs, subcellular membrane markers, including plasma-EGFP, ER-EGFP, and Golgi-EGFP were co-transfected with 2-E plasmids to Vero E6 cells for 24 h. To explore the potential origin of CoV-2- EVs, the Vero E6 cells were transfected with Golgi-EGFP plasmids 48 h after virus infection. After 24-h transfection, cells were fixed in 4% PFA for 7 days and then observed. All of these images and videos were recorded by the Leica TCS-SP8 STED system (Leica Microsystems). The Lenti-mNeonGreen-3×Flag virus was observed in the EGFP channel and 2-E-mCherry was observed in the mCherry channel. The membrane markers including plasma-EGFP, ER-EGFP, and Golgi-EGFP were observed in the EGFP channel. All images shown are representative of at least three randomly selected fields.

### Immunoelectron microscopy

Cells were collected and fixed with 4% (w/v) paraformaldehyde (P6148, Sigma-Aldrich) and 0.2% glutaraldehyde (G5882, Sigma-Aldrich) in 0.1 M PB (pH 7.4) for 30 min at room temperature. Then samples were transferred into 2% PFA and shipped on ice to Peking University for further processing. Cells were incubated in 50 mM glycine in 0.1 M PB two times for 5 min each to block free aldehyde groups. After washing with 0.1 M PB buffer four times for 5 min each, cells were embedded in 2% low gelling temperature agarose (A9414, Sigma-Aldrich) and dissected into 1 mm^3^ blocks. Then blocks were dehydrated through an alcohol series, and embedded in Lowicryl HM 20 (14340, Electron Microscopy Sciences) followed by UV polymerization at –20 °C in a Leica AFS apparatus (Leica Microsystems, Vienna, Austria). Resin blocks were trimmed and sectioned using an ultramicrotome (UC7, Leica Microsystem). Ultrathin sections (75 nm) were collected on nickel grids with a single slot. For immunogold labeling, sections were blocked with blocking buffer (0.5% BSA-c and 0.05% Tween in PBS) for 10 min, and then incubated with primary antibody of SARS-CoV-2 Nucleocapsid Protein (HL344) (1:10, # 26369, Cell Signaling Technology) for 1 h at room temperature. Following several washes in PBS, sections were labeled with protein A Gold 10 nm (1:50; Dept. of Cell Biology, UMC Utrecht, the Netherlands) for 30 min. After washing with PBS, sections were postfixed in 0.1% glutaraldehyde, washed with distilled water, counterstained with 2% uranyl acetate. Grids were viewed on Tecnai G^2^ Spirit BioTWIN electron microscope (FEI) operating at 120 kV. Digital images were photographed with a CCD camera (Orius 832, Gatan).

### Western blot analysis

Proteins were resolved in 12% SDS-PAGE, transferred to PVDF membranes (GE), and incubated with primary antibodies against mCherry (ab125096, Abcam, UK), Flag (AF519, Beyotime, China), hACE-2 (ab108252, Abcam, UK), NRP-1 (ab81321, Abcam, UK), AXL (ab215205, Abcam, UK), TMPRSS2 (ab92323, Abcam, UK) GAPDH (30201ES60, Yeasen, China), N protein (A20021, ABclonal, China), Caspase-3 (14220, CST, USA), Cleaved Caspase-3 (9664, CST, USA), Caspase-7 (12827, CST, USA), Cleaved Caspase-7 (8438, CST, USA), PARP (9542, CST, USA), GSDMD (93709, CST, USA), GSDME (ab215191, Abcam, UK), IL-1β (31202, CST, USA), Caspase-1 (24232, CST, USA), mouse MLKL (AP14272b, Abcepta, USA), mouse pMLKL (Ser345) (D6E3G) (37333, CST, USA), human MLKL (14993, CST, USA), human pMLKL (91689, CST, USA), RIP1 (3493, CST, USA), RIP3 (ab56164, Abcam, UK), GSDMA (ab214818, Abcam, UK), GSDMB (A7474, ABclonal, China), GSDMC (A14550, ABclonal, China). Reactivity was determined using HRP-conjugated secondary antibodies (33101ES60, 33201ES60, Yeasen, China).

### Plaque reduction assay

CoV-2-EVs collected from SARS-CoV-2-infected Vero E6 cells were subjected to three times of freezing and thawing to release the free viral particles. The supernatants were collected after centrifugation at 2000 rpm for 5 min, and diluted as 10^–2^ to 10^–10^. And supernatants collected from Vero E6 and SCC7 cells after CoV-2-EVs infection were diluted as 10^–1^ to 10^–6^. A volume of 200 μL virus diluents was transferred to the monolayer Vero E6 cells in 24-well plate. After incubation for 1 h at 37 °C, the virus diluents were completely removed and cells were washed with PBS followed by 1 mL 0.9% methylcellulose-2% FBS-DMEM culture medium taken to cover the cells. After a 4-day incubation at 37 °C, the medium was removed and cells were fixed with 4% paraformaldehyde then stained with 1% crystal violet. The plaques were manually counted.

### Neutralization test

SARS-CoV-2 (nCoV-2019BetaCoV/Wuhan/WIV04/2019) was preserved at Wuhan institute of virology, Chinese Academy of Sciences. It was propagated and titrated with Vero E6 cells, and its associated operations were performed in a BSL-3 facility. Rabbit neutralizing monoclonal antibody (nAb-1) was purchased from Sino Biological Inc. (China). RBD-specific F(ab’)_2_ (nAb-2) was prepared by our lab and reported previously. nAbs were first diluted with DMEM, and incubated with SARS-CoV-2 (isolate Wuhan-Hu-1) at MOI = 0.01 (~400 PFU) at 37 °C for 1 h. Then the mixture was added to Vero E6 cells seeded in 48-well plate, and the incubation was maintained at 37 °C for 1 h. After that, the mixture was completely removed, cells were covered with 200 μL fresh medium. Twenty-four hours later, 150 μL cell supernatants were taken for viral copy detection as conventional procedure. Non-linear regression curves were analyzed using Prism 8 software to calculate EC_50_ values.

### Antibody escaping and secondary infection

For antibody escaping experiments, CoV-2-EVs were collected and isolated as above. For the first set of experiments, the titers of SARS-CoV-2 free-viruses and CoV-2-EVs-encapsulated viruses were quantified before antibody treatment. In detail, we first quantified the virus copies of free-viruses and vesicle-virus via qRT-PCR. Then, the titer of free viruses was detected. Next, we converted the vesicle-virus copies to titer and got the titer as 1.17 × 10^7^ PFU/mL. Finally, we diluted SARS-CoV-2 and CoV-2-EVs to the equal titer to infect healthy cells. The healthy Vero-E6 cells were infected with SARS-CoV-2 and CoV-2-EVs for 2 h, and then cells were maintained in medium containing two different neutralizing antibodies, nAb-1 (2 μg/mL) and nAb-2 (1 μg/mL). After 24 h, supernatants and cells and partial CoV-2-EVs were collected for viral RNA copy detection. For the second set of experiments, Vero E6 cells were infected with live SARS-CoV-2 virus for 1 h (MOI = 1), and then neutralizing antibodies or control IgG proteins were added. SARS-CoV-2-induced EVs were isolated from the supernatant by differential centrifugation. Naïve Vero E6 cells were incubated with SARS-CoV-2-induced EVs for 24 h and then replaced with fresh medium. Then, the viral copies were tested by qRT-PCR.

For secondary infection experiments, naïve Vero E6/A549/SCC7/H1299/H1299 AXL^–/–^ cells were incubated with CoV-2-EVs for 24 h and then replaced with fresh medium. Vero E6/A549/SCC7/H1299/H1299 AXL^–/–^ cells infected with SARS-CoV-2 at MOI = 0.01 was used as control. Cell supernatants and cells were collected 24 h later for viral RNA copy detection as well.

### Virus loading and qRT-PCR analysis

The cells, supernatants, and CoV-2-EVs were collected as described above. Cell total RNA was extracted using Trizol (Invitrogen, USA). Cell supernatants and CoV-2-EV RNA were isolated with MiniBEST Viral RNA/DNA Extraction Kit (Takara, Japan) as described in the instruction, and cDNA was transcribed with PrimeScript^TM^ RT reagent Kit with gDNA Eraser (Takara, Japan). In detail, 50 μL supernatant was collected for RNA isolation with a MiniBEST Viral RNA/DNA Extraction Kit Ver.5.0 (Takara, AK41820A), and the total RNA was eluted with 30 μL RNase-free water. cDNA was transcribed from 3 μL total RNA in 20 μL reaction system with PrimeScriptTM RT reagent Kit with gDNA Eraser (Takara, AK71648A). Viral copies were quantified from 1 μL cDNA template viral cDNA by a standard curve method on ABI 7500 (Takara TB Green Premix Ex Taq II, AK81975A) with a pair of primers targeting *S* gene. The forward primer (5′-3′) is: CAATGGTTTAACAGGCACAGG; the reverse primer (5′-3′) is: CTCAAGTGTCTGTGGATCACG. The standard curve was set from six points in 20 µL reaction system (2.35 × 10^9^ copies, 2.35 × 10^8^ copies, 2.35 × 10^7^ copies, 2.35 × 10^6^ copies, 2.35 × 10^5^ copies, 2.35 × 10^4^ copies).

### Statistical analysis

All measurements were derived from distinct samples. Statistics were performed in GraphPad Prism. Statistical significance was determined by one-way ANOVA and two-way ANOVA followed by pairwise Student’s *t*-test with Tukey’s or Sidak’s correction. Two-tailed unpaired Student’s *t*-test was performed if only two conditions were compared. Data in the text are presented as means ± SEM. Adjusted *P* values are reported in the figures.

## Supplementary information

Supplementary Information

Supplementary Video S1

Supplementary Video S2

Supplementary Video S3

Supplementary Video S4

Supplementary Video S5
