# Supplementary material for: Extracellular vesicles mediate antibody-resistant transmission of SARS-CoV-2
Source: Cell Discov. 2023 Jan 6;9:2. doi: 10.1038/s41421-022-00510-2 (PMC9821354; doi:10.1038/s41421-022-00510-2)
Supplement: Supplementary file 1 — Supplementary Information [file 41421_2022_510_MOESM1_ESM.pdf]

Supplementary Figures and Videos:

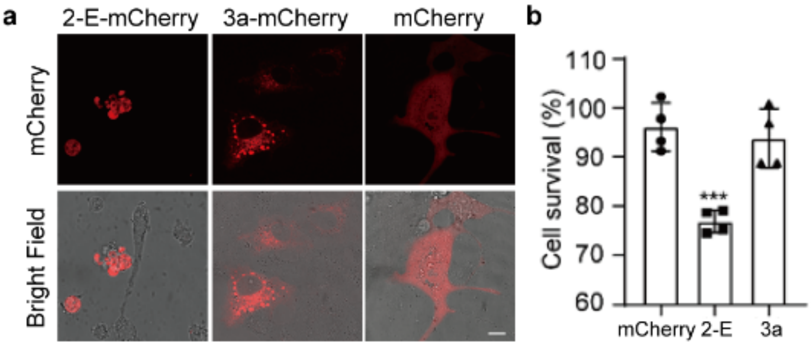

Supplementary Fig. S1: Cell images and viability level after transfection as indicated.

a, Images of Vero E6 after transfected with 2-E channel, 3a channel and mCherry (bar, 10 μm). The controls 3a and mCherry did not induce blebbing and cell death. **b,** Cell survival for Vero E6 cells after transfected with 2-E channel, 3a channel and mCherry for 24 h. Shown were the mean ± SEM of three independent experiments. $^{*}p < 0.05$, $^{**}p < 0.01$, $^{***}p < 0.001$; unpaired Student's t test.

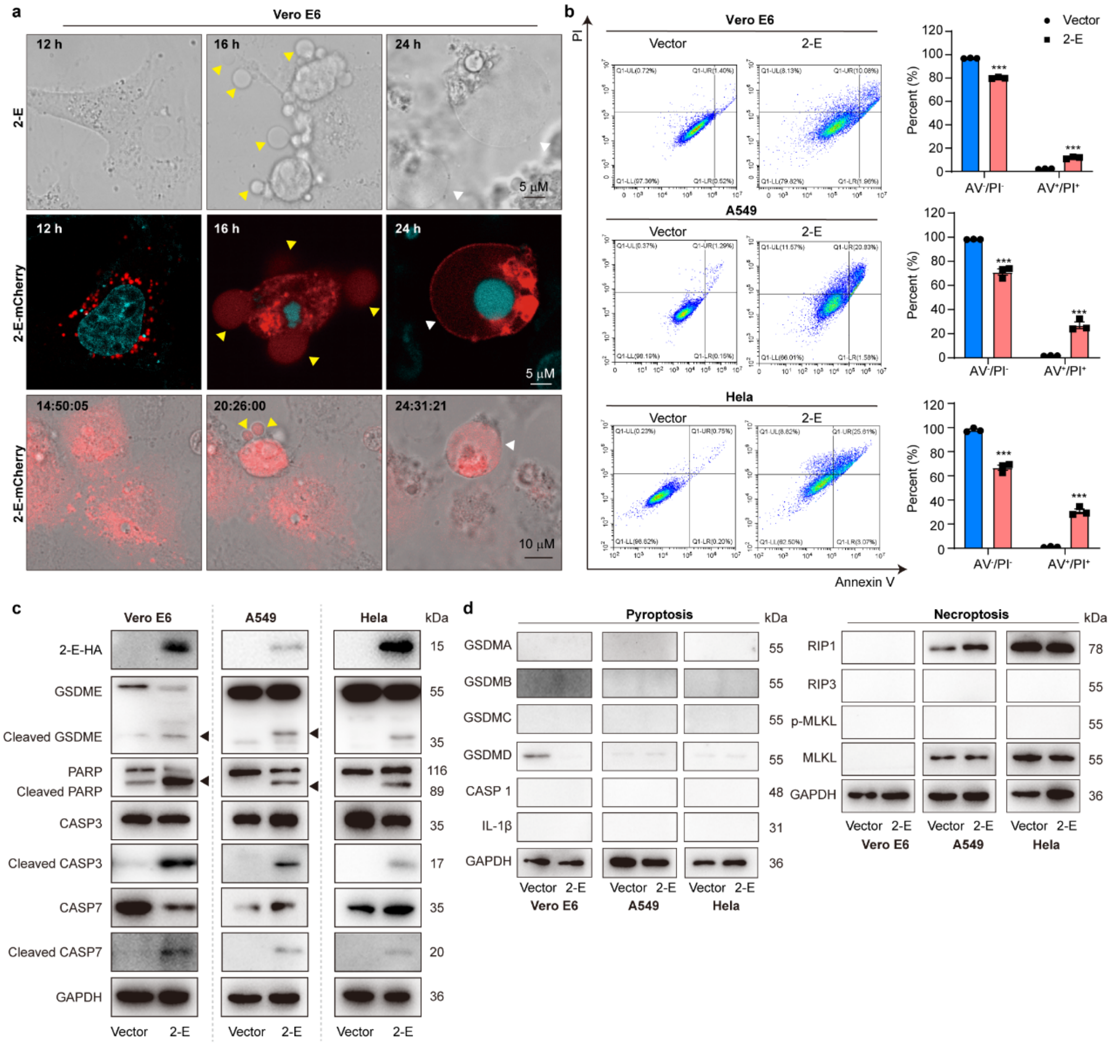

Supplementary Fig. S2: 2-E induces pyroptosis via GSDME cleavage.

a, Images of 2-E expressing Vero E6 cells at different transfection point (up). Fluorescent images of Vero E6 cells after transfection with 2-E-mCherry and stained with Hoechst (middle). Representative time-lapse cell images (fluorescence and bright field) were taken from 12-24 h after 2-E-mCherry transfection (bottom). Arrowheads indicate ballooned pyroptotic cells and secreted vesicles. **b,** Three cell lines transfected with 2-E plasmids respectively. Flow cytometry analysis of Propidium iodide (PI) and Annexin V (AV) stained. **c,** Immunoblotting of 2-E-induced GSDME cleavage by caspase-3 and caspase-7 in three types of cells. **d,** Immunoblotting of cell death pathway

biomarkers in 2-E transfected cells. Shown were the mean ± SEM of three independent experiments. $^{*}p < 0.05$, $^{**}p < 0.01$, $^{***}p < 0.001$; unpaired Student's t test.

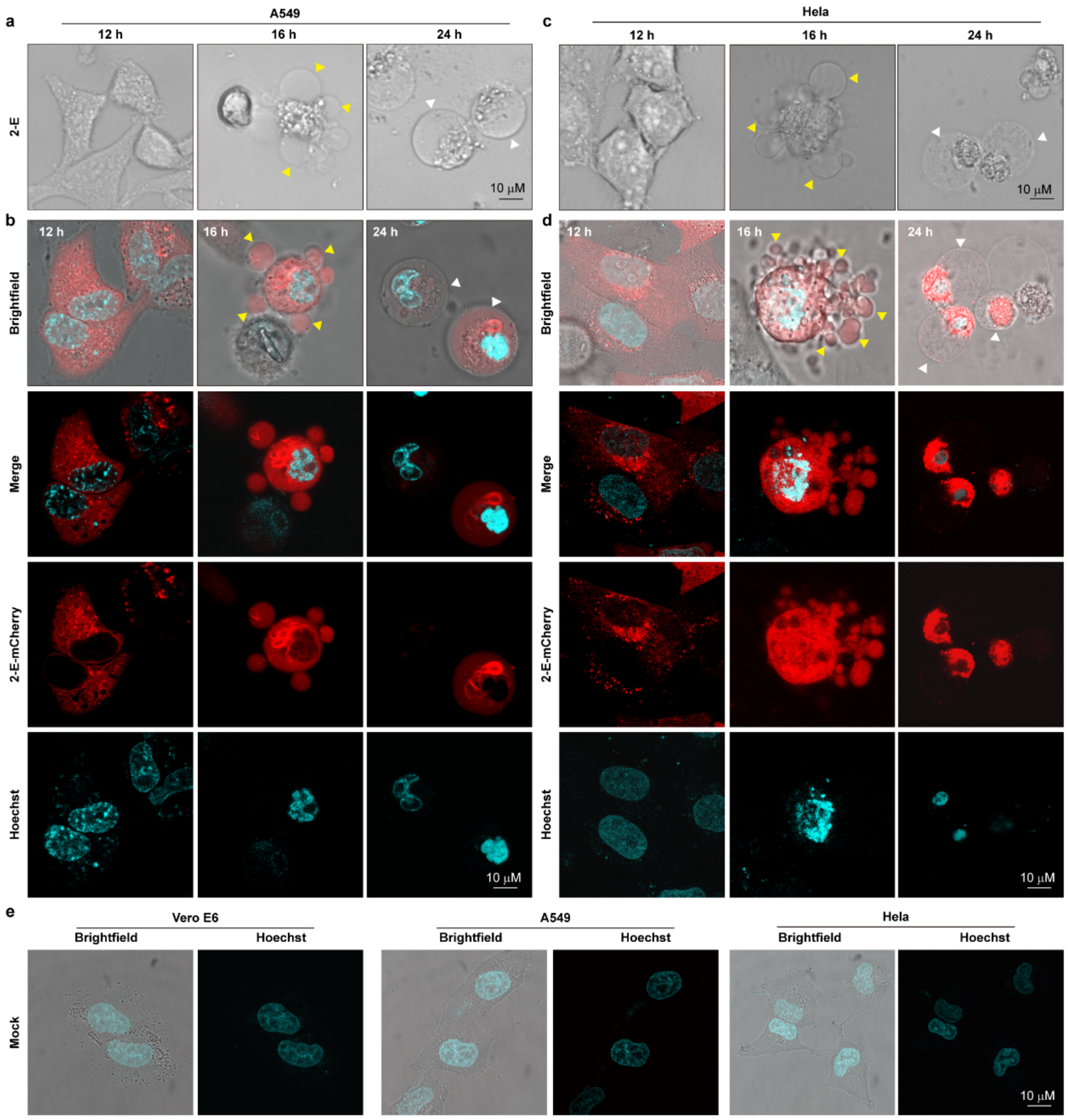

Supplementary Fig. S3: Images of 2-E expression Hela and A549 cell respectively. **a,** Images of 2-E expressing A549 cells at different transfection point. **b,** Representative A549 cell images (fluorescence and bright field) were taken from 12-24 h after 2-E-mCherry transfection. Hoechst staining (Blue) indicates nucleus. Arrowheads indicate ballooned cell membrane characteristic of pyroptotic cells. **c,** Images of 2-E expressing Hela cells at different transfection point. **d,** Representative Hela cell images (fluorescence and bright field) were taken from 12-24 h after 2-E-mCherry transfection. Hoechst staining (Blue) indicates nucleus. Arrowheads indicate ballooned cell

membrane characteristic of pyroptotic cells (a-d). **e,** Images of Vero E6, A549 and Hela cells transfected with vectors as control.

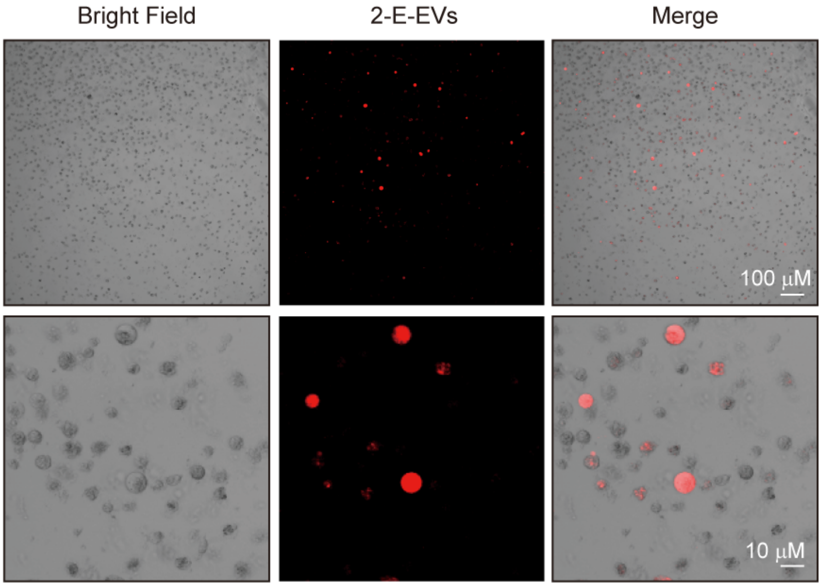

Supplementary Fig. S4: Microscopy images of 2-E-induced EVs. Vero E6 cells transfected with 2-E-mCherry plasmid and 2-E-EVs were isolated from the transfected cells.

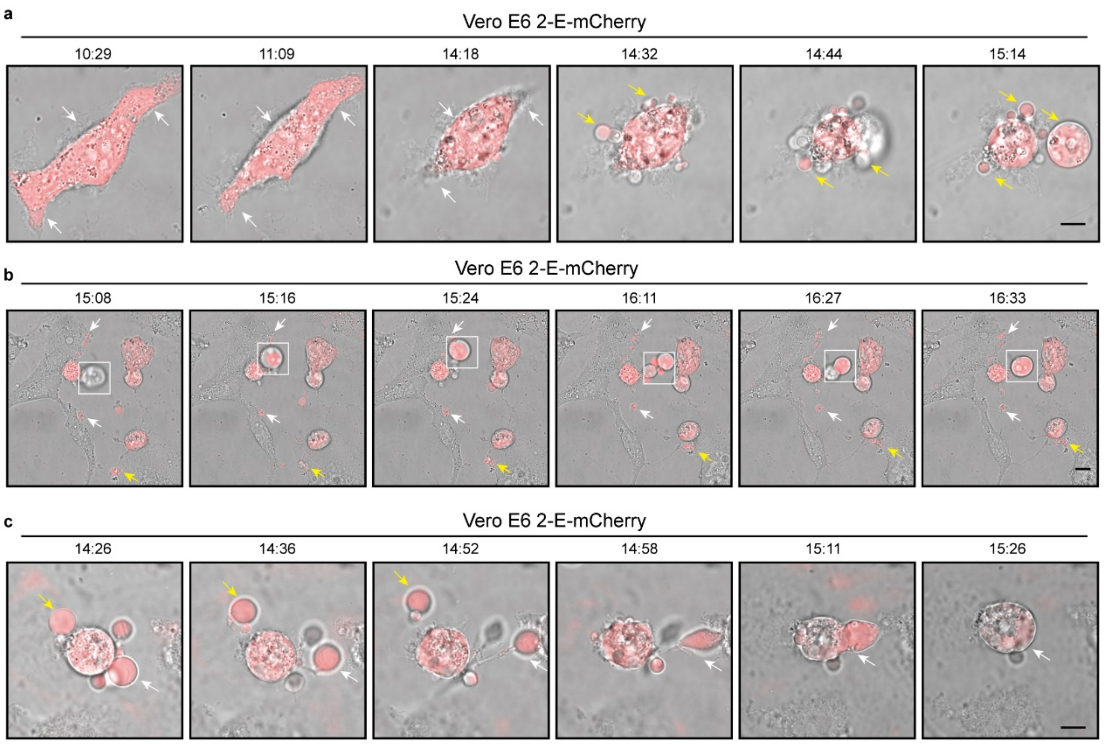

Supplementary Fig. S5: Microscope images of Vero E6 cells transfected with 2-E-mCherry.

a-c, Representative time-lapse cell images (fluorescence and bright field) were taken from 10-16 h after transfection (bar, 10 μm). **a,** White arrow, healthy cell became swelling. Yellow arrow, multiple EVs secreted. **b,** White arrow, attached EVs. Rectangle, fused EVs. Yellow arrows, rope network. **c,** Yellow arrow, the EVs shed from their original cell and floated away. White arrow, the EVs were pulled backed by the elastic rope and fused with its original cell.

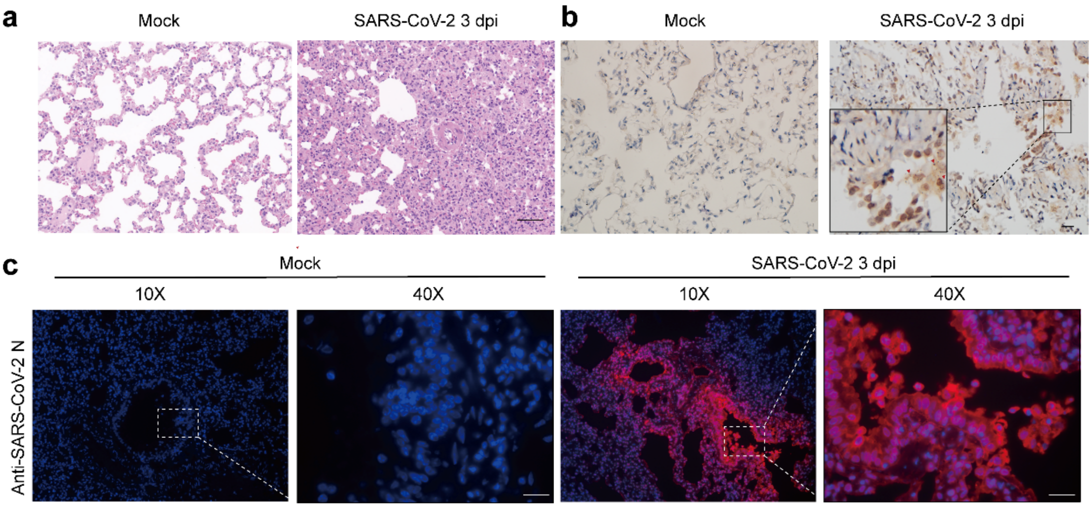

Supplementary Fig. S6: Histopathology of lungs in golden hamster infection models.

a, HE staining mages of golden hamster lung tissues infected with SARS-CoV-2 or not. (bar, 20 μm) **b,** Immunohistochemistry analysis of SARS-CoV-2 N protein in SARS-CoV-2 infected and healthy golden hamster lung tissues (bar, 20 μm). **c,** Immunofluorescent analyzed of SARS-CoV-2. Blue, DAPI; Red, SARS-CoV-2 N protein, which indicated viral infected cells (bar, 20 μm).

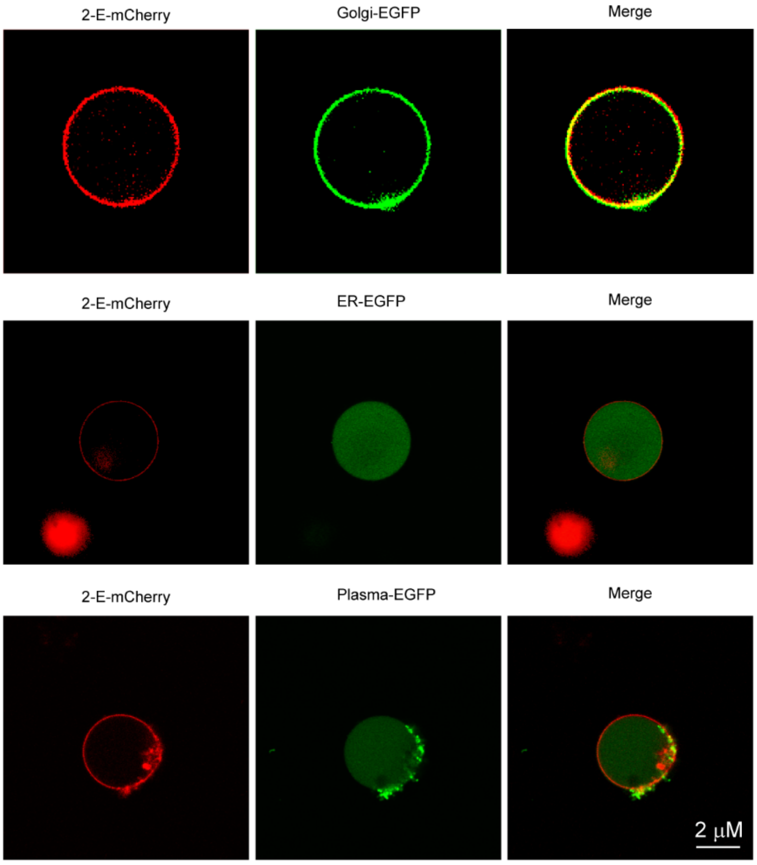

Supplementary Fig. S7: Images of Vero E6 cells after co-transfected 2-E and Golgi-EGFP, ER-EGFP or Plasma-EGFP markers. 2-E-EVs were isolated from the transfected cells.

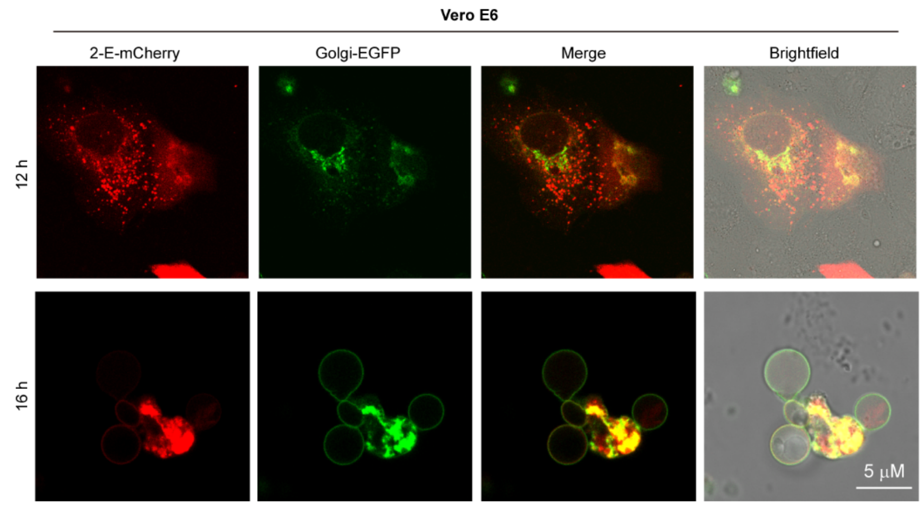

Supplementary Fig. S8: 2-E localizes on Golgi apparatus and forms EVs. Vero E6 cells co-transfected with 2-E-mCherry and Golgi-EGFP (B4GALT1-EGFP, the marker of Golgi). The images were captured at indicated time after transfection.

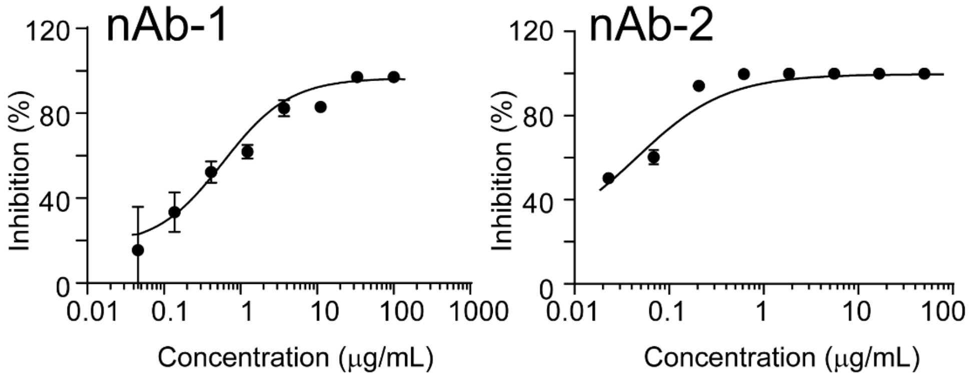

Supplementary Fig. S9: IC_{50}S of two SARS-CoV-2 neutralizing antibodies under 0.01 MOI live virus infection.

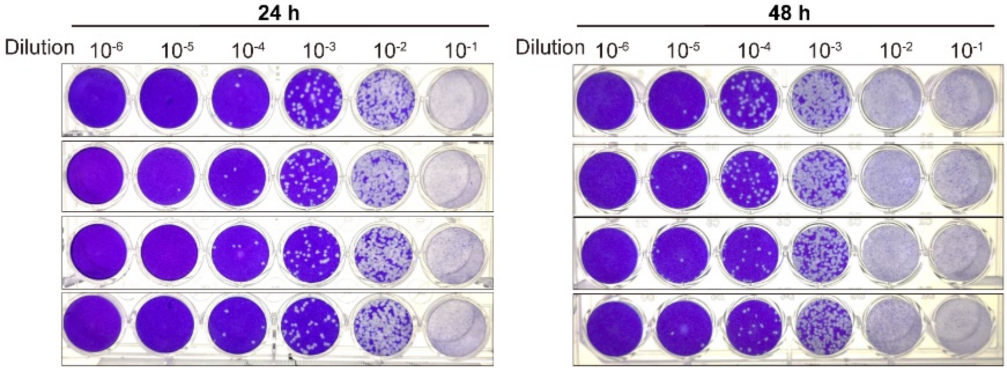

Supplementary Fig. S10: Plaque reduction assay of SARS-CoV-2 induced EVs re-infecting Vero E6 cells for 24 and 48 h.

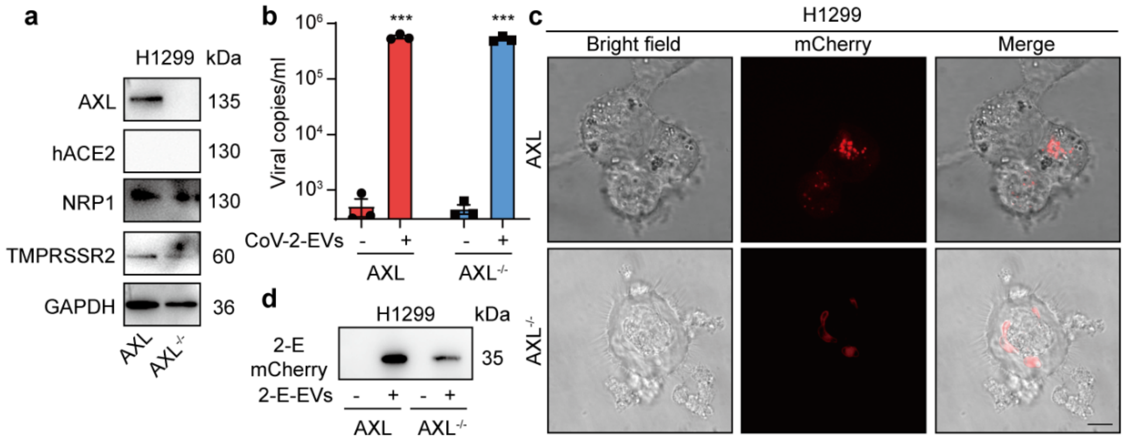

Supplementary Fig. S11: SARS-CoV-2-induced EVs infected H1299 cells independent of AXL receptor.

a, Expression of AXL, hACE-2, NRP-1 and TMPRSSR2 in H1299 and H1299 $AXL^{-/-}$ cells. **b,** H1299 and H1299 $AXL^{-/-}$ cells were incubated with isolated CoV-2-induced EVs and supernatant viral copies was quantified by qRT-PCR (n > 3 independent experiments). **c,** Images of H1299 and H1299 $AXL^{-/-}$ cells that were exposed to 2-E-mCherry-induced EVs (bar, 10 μm). **d,** Immunoblots detection of 2-E-mCherry in the secondary infected cells. H1299 and H1299 $AXL^{-/-}$ cells were exposed to 2-E-mCherry-induced EVs for 24 h. Shown were the mean ± SEM of three independent experiments. $^{*}p < 0.05$, $^{**}p < 0.01$, $^{***}p < 0.001$; unpaired Student's t test.

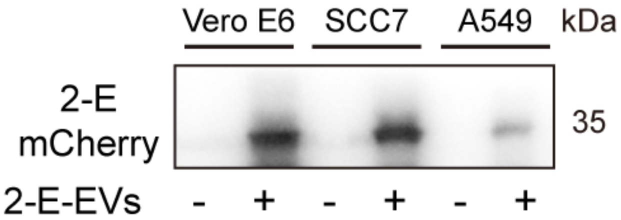

Supplementary Fig. S12: Immunoblots detection of 2-E-mCherry in the secondary infected cells. Vero E6, A549 and SCC7 cells were exposed to 2-E-mCherry-induced EVs for 24 h.

Supplementary Video S1: Representative time-lapse cell images (fluorescence and bright field) were taken from 12-26 h after 2-E-mCherry transfection, related to Supplementary Fig.S2. The morphology of the cell was observed by Leica SP8 STED confocal microscope. Images were taken every 30 s.

Supplementary Videos S2-S3: Representative video of the Vero E6 cells blebbing process after 2-E-mCherry transfection,related to Fig.1d, Supplementary Fig.S5.

Supplementary Video S4: Images of Vero E6 exposed to 2-E-mCherry-induced EVs, related to Fig.5d first line. Naïve Vero E6 cells were exposed to red EVs. The constructed 3D videos supported the presence of intact EVs in cells.

Supplementary Video S5: Images of Vero E6 exposed to 2-E-mCherry-induced EVs, related to Fig.5d second line. Naïve Vero E6 cells were first transfected with EGFP plasmids and then exposed to red EVs. The constructed 3D videos supported the presence of intact EVs in cells.
